# Centenarians’ Experience of the COVID Pandemic in Switzerland

**DOI:** 10.1093/geroni/igab046.419

**Published:** 2021-12-17

**Authors:** Daniela Jopp, Stefano Cavalli, Armin von Gunten, François Herrmann, Carla Gomes Da Rocha, Garnelle Ziade, Kim Uittenhove

**Affiliations:** 1 University of Lausanne, Lausanne, Vaud, Switzerland; 2 University of Applied Sciences and arts of southern switzerland, Manno, Ticino, Switzerland; 3 CHUV - University Hospital of Vaud, Lausanne, Vaud, Switzerland; 4 University of Geneva, Geneva, Geneve, Switzerland; 5 HES-SO Valais Wallis, Sion, Valais, Switzerland; 6 University of Lausanne, lausanne, Vaud, Switzerland; 7 UNIL, Lausanne, Vaud, Switzerland

## Abstract

Being considered as individuals with elevated risk of severe health reactions to the COVID19 infections, governments around the world have put in place wide-ranging measures to protect very old individuals from the virus. In the present study, we investigated centenarians’ experience of the COVID19 pandemic, to reach a better understanding of their vulnerability and resilience. As part of the SWISS100 study, we conducted telephone interviews with 30 centenarians and 40 family members. While almost all centenarians felt not, qualitative data suggested the existence of two groups: One included centenarians lived rather withdrawn and isolated before the crisis and therefore did not experience major changes. The other group included centenarians who suffered substantially from no longer being able to see family and friends and missed valued activities. Family members reported challenges, including centenarians’ decline in mental and physical health. Findings highlight the importance of different vulnerability profiles and lock-down side effects.

